# Prevalence of Non-responders for Blood Pressure and Cardiometabolic Risk Factors Among Prehypertensive Women After Long-Term High-Intensity Interval Training

**DOI:** 10.3389/fphys.2018.01443

**Published:** 2018-10-23

**Authors:** Cristian Álvarez, Rodrigo Ramírez-Campillo, Carlos Cristi-Montero, Robinson Ramírez-Vélez, Mikel Izquierdo

**Affiliations:** ^1^Laboratory of Human Performance, Quality of Life and Wellness Research Group, Department of Physical Activity Sciences, Universidad de Los Lagos, Osorno, Chile; ^2^IRyS Group, Pontificia Universidad Católica de Valparaíso, Valparaíso, Chile; ^3^Centro de Estudios en Medición de la Actividad Física (CEMA), Escuela de Medicina y Ciencias de la Salud, Universidad del Rosario, Bogotá, Colombia; ^4^Department of Health Sciences, Public University of Navarra, Navarrabiomed, CIBER of Frailty and Healthy Aging (CIBERFES), Instituto de Salud Carlos III, Pamplona, Spain

**Keywords:** high-intensity interval training, prehypertension, responders, non-responders, women, risk factors, systolic blood pressure

## Abstract

**Background:** Exercise is known to improve cardiometabolic outcomes; however, results are typically reported as mean values, and there is wide interindividual variability in terms of response that has not been explored in populations at risk for hypertension. Our aim was to investigate both the effects on and the prevalence of non-responders (NRs) for decreasing blood pressure (BP) and other risk factors among prehypertensive women after long-term high-intensity interval training (HIIT). A secondary aim was to report potential variables that can predict decreases in BP after HIIT.

**Methods:** Sedentary overweight/obese women (age 35.9 ± 5.4 year; body mass index [BMI] 30.9 ± 6.2 kg/m^2^) were assigned to a prehypertensive (PreHTN; *N* = 44) or normotensive (NT; *N* = 40) group according to their ambulatory BP at baseline. Subjects underwent a thrice-weekly 16-week HIIT program (7–10 × 1 min exercise with 2 min of rest). Training-induced changes in body composition and cardiovascular, metabolic, strength, and endurance performance markers were measured, and the prevalence of NRs was reported as a percentage. All outcomes were analyzed by multivariable regression.

**Results:** Statistically significant (*P* < 0.05) decreases in systolic BP (SBP) were detected in the PreHTN group (Δ −8 mmHg) compared with baseline, whereas the NT group (Δ + 3 mmHg) showed a non-significant increase in SBP. Diastolic BP (DBP) was significantly decreased in the PreHTN group (Δ −5.8 mmHg) and non-significantly decreased (Δ −2 mmHg) in the NT group. Also, there were significant differences (*P* < 0.0001) in the prevalence of NRs based on SBP between the PreHTN and NT groups (11.4 vs. 68.8%), but similar prevalence of NRs based on DBP. SBP alone was a powerful predictive factor for a beneficial SBP reduction, explaining 51.2% of the results, which was similar to other more complex models tested.

**Conclusion:** The prevalence of NRs based on SBP and DBP was different between prehypertensive and normotensive subjects after 16 weeks of HIIT. Other comorbidities such as body composition and metabolic outcomes showed almost similar modifications between prehypertensive and normotensive subjects, being the most basic predictive factor for BP reduction baseline SBP, which we refer to as ‘BP health status’ (51.2%). This improvement in BP was accompanied by other known improvements of HIIT on body composition, metabolic and endurance performance in both study cohorts.

**Trial Registration:** ClinicalTrials.gov ID: NCT03000140 (Register 20 December, 2016).

## Introduction

The prehypertensive state is generally associated with physical inactivity (not engaged in physical activity according to international physical activity guidelines) (O’Donovan et al., 2010) and other risk factors including diet, sodium intake, and smoking, which is the most important contributor to hypertension (HTN) development (Díaz-Martínez et al., 2017). HTN is also the most common primary diagnosis in Chile, and has increased in adult women from 25.0% in 2009–2010 to 27.7% in 2017 (Hawley, 2009). Because a single session of endurance exercise decreases 24-h blood pressure (BP) (Karoline de Morais et al., 2015), chronic exercise (i.e., regular exposure to exercise) provides powerful benefits for prehypertensive subjects (Park et al., 2006), where the changes (i.e., decreases) of blood pressure in normotensive subjects have been regularly reported. These effects have been corroborated by meta-analyses and, accordingly, prolonged endurance training has been recognized as a strategy to decrease BP and other comorbidities in prehypertensive and hypertensive populations (Montero et al., 2014).

Unfortunately, while there is ample evidence to support that exercise training (e.g., endurance exercise) decreases BP (Pescatello et al., 2015), ‘lack of time’ is frequently cited by the sedentary population as the main barrier to adherence to exercise guidelines (i.e., less than 150 min of low-moderate-intensity exercise/week or 75 min of vigorous-intensity exercise/week) (Trost et al., 2002). Therefore, more research in exercise science is needed for the prevention of HTN and the management of both early prehypertension and common comorbidities including obesity (e.g., waist circumference or fat mass) and dyslipidemia (i.e., to decrease low-density lipoprotein or triglycerides) (Russo et al., 2018). In this regard, high-intensity interval training (HIIT, defined as repeated bouts of high-intensity exercise interspersed with rest periods) has been reported to be a powerful regimen for improving body composition and cardiovascular, metabolic, and performance variables (Gibala et al., 2012). HIIT also reduces both arterial stiffness and microvascular dysfunction in hypertensive individuals (Shrout et al., 2017), and increases the expression of key proteins such as peroxisome proliferator-activated receptor-γ coactivator 1α and vascular endothelial growth factor, that play an important role in arterial remodeling, in trained/healthy subjects (Lim et al., 2012). Moreover, when compared with endurance exercise, HIIT has been shown to improve endothelial function in subjects at risk for HTN (Guimaraes et al., 2010) and also in patients with metabolic syndrome (Tjønna et al., 2008). Despite these beneficial effects from HIIT, it remains unknown whether HIIT impacts BP and comorbidities associated with the prehypertensive state more than normotensive subjects.

Most exercise intervention studies report mean data; however, there is usually wide interindividual variability (Bouchard and Rankinen, 2001). This implies that under the same stimulus, some subjects, referred to as responders (Rs), may achieve benefits, while others, referred to as non-responders (NRs), may exhibit a worsened or unchanged response (Bonafiglia et al., 2016). Although genetic [i.e., some polymorphisms (Yako et al., 2018)] and environmental (regular exercise, diet, and sleep) factors have been described to predictive Rs and NRs (Mann et al., 2014), not all of these factors have been explored in detail, such as the effects of a different ‘BP health status’ (e.g., prehypertension vs. HTN) on the prevalence of NRs after training. For example, it has been widely known that individuals with HTN show power and clinical decreases in blood pressure after traditional endurance exercise training (Cade et al., 1984). A recent report showed that after 6 weeks of HIIT ∼60% of participants were NRs for a decrease in diastolic BP (DBP) (Higgins et al., 2015). By contrast, after 20 weeks of endurance training a minority of subjects were NRs (12.2%), without any decreases in systolic BP (SBP) (Bouchard et al., 2012). Additionally, when prehypertensive individuals underwent 6 months of different training regimens [i.e., endurance, resistant, or concurrent training (i.e., endurance plus resistant training)], ∼60% were NRs for decreases in SBP or DBP (Moker et al., 2014).

Determining both the effects on and prevalence of NRs after HIIT is important for choosing the appropriate exercise regimen and optimizing responses in different cohorts (e.g., athletes or individuals with risk factors for HTN). Furthermore, knowing which variables can predict more changes after training could be useful to more efficiently determine the time to invest in a particular exercise modality. Along this line, some authors have shown that the magnitude of acute changes from chronic exercise is a good predictor of improvements in metabolism (Hecksteden et al., 2013a) and decreases in BP (Hecksteden et al., 2013b). However, neither of these studies simultaneously reported the effects on and the prevalence of NRs, nor determined the variables that can predict responses in populations at risk for HTN. Several studies have reported that certain factors such as exercise modality (Álvarez et al., 2017), acute exercise (Hecksteden et al., 2013b), baseline differences in aerobic fitness (Bouchard and Rankinen, 2001), or metabolic health status (i.e., healthy or diagnosed with a metabolic disease) (Alvarez et al., 2017) can influence the prevalence of NRs, but all have been conducted mainly with normotensive cohorts. Thus, due to the lack of evidence on the effects on and prevalence of NRs after HIIT among prehypertensive than normotensive subjects, and on the potential predictive factors for lowering BP, the aim of the present study was to investigate both the effects on and the prevalence of NRs for decreased BP and comorbidities among prehypertensive women after HIIT. A secondary aim was to report potential variables that predict decreases in BP. Based on previous genetic studies, where the training variation can be attributed to multiple factors, and considering a non-epidemiological sample size (Mann et al., 2014), we hypothesized that independent of training-induced changes there would be a similar effect from HIIT and a similar prevalence of NRs between prehypertensive and normotensive subjects after a 16-week HIIT intervention.

## Materials and Methods

### Subjects

We studied overweight or obese sedentary women [body mass index (BMI) between 25 and 30 kg/m^2^; aged 30–40 years] who had been diagnosed with prehypertension for at least 1 month but no more than 3 months by our research team. The study inclusion criteria were as follows: (a) ambulatory SBP >120 and <140 mmHg and/or DBP ≥80 and <90 mmHg, according to standard classifications of blood pressure (Pescatello et al., 2015); (b) lack of drug therapy in the previous 3 months; (c) BMI >24 and <35 kg/m^2^; (d) physical inactivity (i.e., <150 min of low/moderate or <75 min of moderate/vigorous physical activity/week (O’Donovan et al., 2010), as assessed by the International Physical Activity Questionnaire previously validated in the Chilean population) (Seron et al., 2010); and (e) independent of commonly altered metabolic variables (normocholesterolemic/or slightly hypercholesterolemic) [total cholesterol (TC) <200/≥200 mg/dL, low-density lipoprotein cholesterol (LDL-C) <140/≥140 mg/dL, high-density lipoprotein cholesterol (HDL-C) ≤30/>30 mg/dL, and triglycerides (TG) ≤150/>150 mg/dL] following standard classifications (Turnbull et al., 2005). All subjects were not under pharmacological therapy. Subjects with (a) cardiovascular contraindications to exercise; (b) histories of stroke; (c) asthma or chronic obstructive pulmonary disease; (d) musculoskeletal disorders such as muscle or back pain; and (e) a history of smoking in the last 3 months were excluded. A minimum compliance to the exercise program of 70% was required for patients in the intervention group to be included in the final statistical analysis. The trial is registered on ClinicalTrials.gov; ID: NCT03000140 (registered 20 December 2016).

**FIGURE 1:**
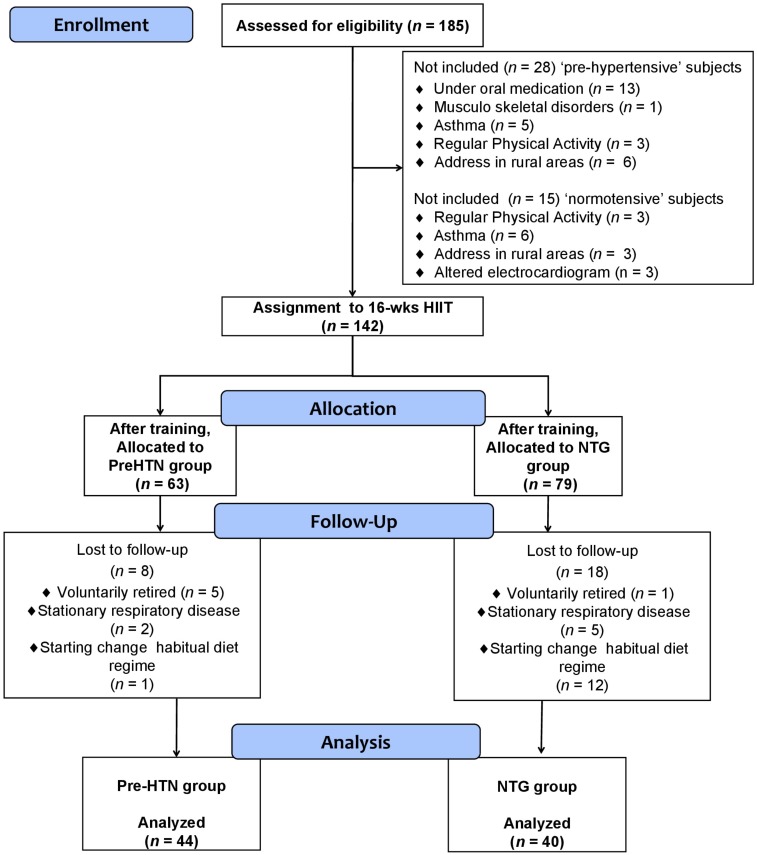
Study design.

**FIGURE 2:**
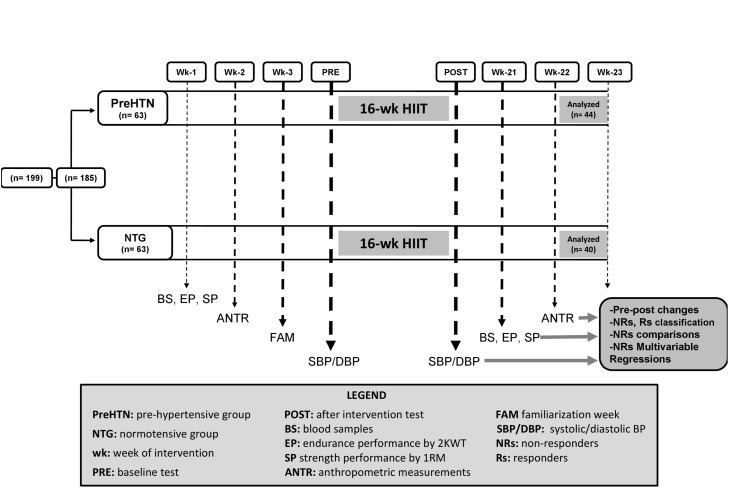
Study protocol.

One hundred and ninety-nine healthy and prehypertensive subjects (aged 25 to 40 years) from the Family Healthcare Center Tomas Rojas of Los Lagos (Chile) were invited to participate by phone; these participants were provided with explanations about the study aims (first stage), informed about the study and invited to be formally screened. Subsequently, one hundred and eighty-five (*N* = 185) subjects agreed to participate in the second stage of screening, the first baseline measurements and the third stage of education regarding the experimental procedures for exercise training. The subjects underwent a structured medical history, medical record review, and physical examination by a physician to assess eligibility based on the criteria. All participants underwent 16 weeks of HIIT by cycling on ergometers and were statistically analyzed as two non-randomized groups: the prehypertensive (PreHTN) and normotensive (NTG) groups. After the intervention period, forty-four subjects were included in the final sample (PreHTN, aged 35.2 ± 5.1 years; BMI 31.9 ± 5.8 kg/m^2^; *N* = 44), and forty subjects were included in the normotensive group (NTG, aged 36.6 ± 5.8 years; BMI 30.0 ± 6.6 kg/m^2^; *N* = 40). The study design is shown in Figure 1, and the study protocol is shown in Figure 2.

### Classification of Responders (Rs) and Non-responders (NRs)

To classify the participants as Rs and NRs based on a decrease in SBP/DBP or the other dependent covariables, the typical error (TE) was calculated, similar to recent studies (Bonafiglia et al., 2016), using the following equation:

$$\mathrm{TE}={\mathrm{SD}}_{\mathrm{diff}}/\sqrt{2}$$

where *SD_diff_* is the variance (standard deviation) of the different scores observed between the two repeats of each test. NRs were defined as an individual who failed to demonstrate a decrease or increase (in favor of beneficial changes) that was greater than two times the TE away from zero. A change beyond two times the TE indicates that there is a high probability (i.e., 12 to 1 odds) that this response is a true physiological adaptation beyond what might be expected to result from technical and/or biological variability (Hopkins, 2000). Thus, the cutoff values were the following for body mass, 2 × TE = 0.500 kg; BMI, 2 × TE = 0.21 kg/m^2^; waist circumference, 2 × TE = 1.1 cm; tricipital skinfold, 2 × TE = 1.3 mm; suprailiac skinfold, 2 × TE = 2.6 mm; abdominal skinfold, 2 × TE = 2.9 mm; fat mass, 2 × TE = 3.8%; muscle mass, 2 × TE = 0.3 kg; systolic BP, 2 × TE = 8.0 mmHg; diastolic BP, 4.9 mmHg; heart rate at rest, 2 × TE = 5.4 beats/min; fasting glucose, 2 × TE = 4.5 mg/dL; TC, 2 × TE = 7.1 mg/dL; LDL-C, 2 × TE = 5.2 mg/dL; HDL-C, 2 × TE = 3.9 mg/dL; TG, 2 × TE = 14.6 mg/dL; 1RM_LE_, 2 × TE = 5.0 kg; and 2KMWT, 2 × TE = 1.5 min/s.

### HIIT Program

Before the intervention, all subjects were familiarized with the HIIT program over three sessions. The participants underwent a thrice-weekly progressive program for 16 weeks. All exercise sessions were performed on cycle ergometers (OXFORD^*TM*^, model BE2601, OXFORD Inc., Santiago, Chile) and were supervised by an exercise physiologist. The HIIT program consisted of high-intensity intervals of work (cycling) for 1 min at a subjective intensity of 8–10 points on the modified Borg scale, which ranges from 1–10 points, similar to other reports (Álvarez et al., 2017) separated by an inactive (no movement on the bicycle) recovery period of 2 min. The training sessions were structured according to the following progression (presented in time cycling/rest/repetitions): weeks 1–4: 1/2/7; weeks 5–8: 1/2/8; weeks 9–12: 1/2/9; and weeks 13–16: 1/2/10. The total range of time investment was as follows: weeks 1–4, 21 min; and weeks 13–16, 30 min/session). Heart rate was continuously monitored among the subjects (ProTrainer 5, Polar Electro, Inc., Kempele, Finland), and their efforts were adjusted to maintain cycling at the subjective effort proposed. Thus, when a subject reported starting the first interval of cycling at 8–10 points on the Borg scale (corresponding to a 100-watt load, for example), this level was rechecked at each of the 3 sessions, and it was usually necessary to increase the load (watts) of cycling to maintain an initial intensity of cycling at 8–10 points, according to the normal exercise adaptations to a new threshold. This subjective intensity corresponded with all intervals of work to a range from 70–100% of the maximum heart rate based on age.

### Exercise Training Compliance

Compliance indices for attendance were calculated for the groups by dividing the total number of sessions attended by the number of training sessions prescribed in percentages. Intensity adherence was calculated from the mean 8 ± 2 points on the Borg modified scale (1–10) based on the cycling ratings across all exercises and sessions for weeks 2–16. The results showed that exercise compliance was 85.1% (41 sessions) in the PreHTN group and 93.7% (45 sessions) in the NTG, and there were no significant differences between groups.

### Assessments

#### Body Composition Assessment

For three sessions prior to the start of the pediment, participants were familiarized with the tests, and 1 week before and after the 16-week follow-up, anthropometric, cardiovascular, metabolic, and performance measurements were obtained. Body mass (kg) was measured (to the nearest 0.1 kg) using a professional scale (Health o Meter^*TM*^ Professional, Sunbeam Products, Inc., Chicago, IL, United States). Height (m) was assessed using the same machine to the nearest 0.1 m of accuracy, similar to previous studies (Jebb et al., 2007). BMI was calculated as body mass divided by height squared (kg/m^2^). Waist circumference (cm) was measured to the nearest 0.1 cm using a flexible and unextendible measuring tape (Hoechstmass^*TM*^, Sulzbach, Germany). Four skinfold thickness measurements were obtained (tricipital, suprailiac, subscapular, and abdominal) using a Langue^*TM*^ caliper (Beta Technology, Inc., Santa Cruz, CA, United States) by a professional, and the same evaluator made the measurements in both the pre- and posttest stages following the standard protocols (Marfell-Jones et al., 2006). The percent (%) fat mass and % muscle mass were assessed by bioimpedance using a digital scale (Omron HBF-INT^*TM*^, Omron Healthcare, Inc., Lake Forest, IL, United States). This procedure was conducted without metal or watches on the body to increase precision, and the average of three measurements was used.

#### Cardiovascular Health Assessment

Ambulatory SBP and DBP were measured using an automatic monitor (Omron^*TM*^ HEM 7114^*TM*^, Omron Healthcare, Inc., Lake Forest, IL, United States) in triplicate (2-min interval between measurements) with subjects in a seated position after they had rested for 15 min. The average data were recorded for each participant based on standard classification procedures (Mancia et al., 2013). Heart rate at rest was measured using a monitor (ProTrainer 5^*TM*^, Polar Electro Inc., Kempele, Finland) after at least 15 min of rest.

#### Strength Performance Assessment

Strength performance was assessed 1 week before and after the intervention using the one-repetition maximum leg extension (1RM_LE_) test, which was implemented according to similar procedures previously described (Álvarez et al., 2017). The 1RM_LE_ was performed using an exercise machine (OXFORD^*TM*^, model EE4002, Santiago, Chile) in the morning between 9 and 11 o’clock. The highest load of three attempts was reported as an average value.

#### Endurance Performance Assessment

On the third day, after 2 days of familiarization, the endurance performance test was conducted. The test involved a 2-km walking test (2KMWT) (Álvarez et al., 2017) in an indoor sports court (100-m track) after a 10-min warm-up of walking at low intensity and performing slow movements involving the knee and ankle joints. The subjects were instructed to walk as fast as possible with a steady pace and were warned not to run. Heart rate was continuously monitored (ProTrainer 5^*TM*^, Polar Electro Inc., Kempele, Finland) during the test. To ensure an accurate test, participants were encouraged to walk faster if their heart rate was lower than 70% of the maximum heart rate based on age. The time spent on the 2KMWT and the average heart rate during the 2KMWT were registered and used for the analysis.

### Blood Analyses

Blood samples (4 ml) were collected before and after the 16-week follow-up in the morning and after a 10-h overnight fast. Posttraining blood sampling of the subjects was performed at least 48 h after the last exercise session to avoid any acute effects of exercise. The samples were placed on ice and centrifuged at 4000 rpm for 5 min at 4°C. Plasma samples were immediately transferred to prechilled microtubes and stored at −20°C for subsequent analysis. Plasma glucose was analyzed by enzymatic methods using standard kits (Wiener Lab, Inc., Rosario, Argentina) and an automatic analyzer (Metrolab 2300 Plus^*TM*^, Metrolab Biomed, Inc., Buenos Aires, Argentina). TC, HDL-C and TG were analyzed using an enzymatic calorimetric method (Diagnostica mbH, Alemania). LDL-C was calculated using the Friedewald equation (Friedewald et al., 1972).

### Statistical Analyses

Data are presented as the mean ± standard deviation (SD). Normality and homoscedasticity assumptions for all data were checked using the Shapiro-Wilk and Levene tests. Wilcoxon’s test was used for non-parametric data. Student’s *t*-test was performed to test for differences between groups at baseline and for each delta between groups. In addition, the non-parametric Wilcoxon test was applied for variables with non-normal distribution (TC and 2KMWT). To reduce within-group variability, a univariate test (ANCOVA) was performed for SBP and DBP as main variables using anthropometric covariables. Repeated-measures ANOVA was used to make comparisons based on group and time. After the intervention, delta values (Δ) were calculated in each dependent variable. Subjects were categorized as Rs and NRs according to the previously mentioned criteria (Hopkins, 2000). Bonferroni *post hoc* test was applied to determine the differences between groups. To test for differences between the prevalence of NRs in the PreHTN group and NTG, the Chi-Square test (X^2^) was used for categorical variables. The odds ratios (ORs) of NRs to HIIT were applied for both NR variables between groups, with an OR ≥2 indicating a high risk of being an NR. Finally, five different models (Model 1, based on only SBP at baseline; Model 2, based on SBP at baseline plus body composition changes; Model 3, based on SBP at baseline plus body composition and cardiovascular changes; Model 4, based on SBP at baseline plus body composition, cardiovascular, and metabolic changes; and Model 5, based on SBP at baseline plus body composition, cardiovascular, metabolic, and performance changes) were applied in order to predict the SBP changes. All statistical analyses were performed with SPSS software version 18 (SPSS^*TM*^ Inc., Chicago, IL, United States). The alpha level was fixed at *P* < 0.05 for all tests of statistical significance.

## Results

### Baseline Measurements

As expected based on the study design, there were significant differences in SBP (128 ± 6 vs. 108 ± 5 mmHg, *P* = 0.003) and DBP (85 ± 8 vs. 75 ± 10 mmHg, *P* = 0.007) at baseline between the PreHTN group and NTG (Table 1). There were no baseline differences in the other dependent anthropometric, cardiovascular, metabolic and performance covariables between groups (Table 1).

**Table 1:** Characteristics of the sample before and after the 16-weeks follow-up.

	PreHTN Pre	PreHTN Post	Δ	NTG Pre	NTG Post	Δ	PreHTN vs. NTG Baseline^†^	ΔPreHTN vs. ΔNTG Pre-post^†^
*n*	44			40				
Age (y)	35.2 ± 5.1			36.6 ± 5.8			0.572	
Height (m)	1.58 ± 5.6			1.59 ± 6.1				
Time elapsed from diagnosis (y)	1.5			1.2				
**Anthropometry**								
Body mass (kg)	79.8 ± 14.8	76.4 ± 14.1^∗∗∗^	−3.3 ± 2.9^&^	75.2 ± 12.3	73.2 ± 11.8^∗∗∗^	−2.0 ± 2.5	*P* = 0.131	*P* = 0.091
Body mass index (kg/m^2^)	31.9 ± 5.8	30.5 ± 5.7^∗∗∗^	−1.4 ± 1.0	30.0 ± 6.6	29.5 ± 6.4^∗^	−0.5 ± 1.9	*P* = 0.182	*P* = 0.167
Waist circumference (cm)	101.5 ± 11	98.5 ± 12^∗∗∗^	−2.9 ± 5.1^&^	98.0 ± 10.7	93.8 ± 10.3^∗∗∗^	−4.2 ± 2.9	*P* = 0.145	***P* = 0.037**
Tricipital skinfold (mm)	26.7 ± 8.2	22.1 ± 6.6^∗^	−4.6 ± 5.5	24.9 ± 7.2	20.1 ± 8.5^∗^	−4.8 ± 5.1	*P* = 0.234	*P* = 0.233
Supra-iliac skinfold (mm)	34.5 ± 9.1	27.2 ± 8.8^∗∗^	−7.3 ± 5.4	32.5 ± 6.2	26.9 ± 7.3^∗∗^	−5.6 ± 5.0	*P* = 0.189	*P* = 0.246
Abdominal skinfold (mm)	44.9 ± 8.1	33.4 ± 9.8^∗∗∗^	−11.5 ± 6.3^¥^	43.1 ± 7.2	35.5 ± 4.1^∗∗∗^	−7.6 ± 5.9	*P* = 0.156	***P* < 0.001**
Fat mass (%)	42.3 ± 6.8	36.5 ± 8.1^∗∗∗^	−5.8 ± 6.2	40.6 ± 7.1	36.8 ± 8.4^∗∗^	−3.8 ± 5.5	*P* = 0.138	*P* = 0.110
Muscle mass (%)	21.3 ± 7.5	21.5 ± 9.9	+0.2 ± 1.5	22.4 ± 5.9	22.7 ± 9.4	+0.3 ± 1.7	*P* = 0.119	*P* = 0.188
**Cardiovascular**								
Systolic blood pressure (mmHg)	128.0 ± 6.0	120.0 ± 6.0^∗∗∗^	−8.0 ± 7.0^$^	108.0 ± 5.0	111.0 ± 8.0	+3.0 ± 9.8	***P* = 0.003**	***P* < 0.0001**
Diastolic blood pressure (mmHg)	85.0 ± 8.0	80.0 ± 10.0^∗∗∗^	−5.8 ± 11.7^¥^	75.0 ± 10.0	73.0 ± 10.0	−2.0 ± 4.9	***P* = 0.007**	***P <* 0.001**
Heart rate rest (beats/min)	84.0 ± 6.0	80.0 ± 8.0^∗∗∗^	−4.0 ± 6.0	82.0 ± 6.0	80.0 ± 8.0	−2.0 ± 5.0	*P* = 0.078	*P* = 0.079
**Metabolic**								
Fasting glucose (mg/dL)	98.0 ± 9.0	95.0 ± 8.0^∗^	−3.3 ± 9.0^¥^	95.0 ± 9.0	90.0 ± 7.0^∗∗^	−5.0 ± 5.8	*P* = 0.078	***P <* 0.01**
Total cholesterol (mg/dL)	190.0 ± 43.0	182.0 ± 33.0	−8.0 ± 25.2	194.0 ± 32.0	180.0 ± 20.0^∗∗^	−14.0 ± 29.6	*P* = 0.652^‡^	*P* = 0.062^‡^
Low-density lipids (mg/dL)	109.0 ± 37.0	106.0 ± 32.0	−2.6 ± 18.7^¥^	120.0 ± 27.0	110.0 ± 20.0^∗∗^	−10.0 ± 21.7	*P* = 0.114	***P* < 0.01**
High-density lipids (mg/dL)	48.0 ± 11.0	53.0 ± 10.0^∗∗^	5.0 ± 6.6	53.0 ± 10.0	54.0 ± 9.0	+1.0 ± 10.1	*P* = 0.076	*P* = 0.221
Triglycerides (mg/dL)	130.0 ± 61.0	117.0 ± 45.0^∗^	−13.9 ± 30.9^&^	133.0 ± 50.0	111.0 ± 32.0^∗∗∗^	−22.0 ± 36.4	*P* = 0.132	***P* = 0.045**
***Strength performance***								
1RM_LE_ (kg)	36.0 ± 7.0	39.0 ± 9.0	+3.0 ± 4.0	33.0 ± 7.0	35.0 ± 8.0	+2.0 ± 6.0	*P* = 0.088	*P* = 0.153
***Endurance performance***								
2KMWT (min.s)	23.47 ± 3.2	19.33 ± 3.3^∗∗∗^	−3.14 ± 3.44	23.11 ± 4.4	20.18 ± 4.5^∗∗∗^	−3.34 ± 3.41	*P* = 0.783^‡^	*P* = 0.328^‡^

*Results presented as mean ± SD. PreHTN, pre hypertensive group; NTG, normotensive group; Δ, delta pre-post changes according to each biological unit of assessment; 1RM_LE_, one maximum repetition of leg-extension strength test; 2KMWT, 2 kilometers walking test. ^∗^P < 0.05, ^∗∗^P < 0.001, ^∗∗∗^P < 0.0001 post vs. pre intervention within-group. ^†^Compared by the Student t-test. ^‡^Compared by the Wilcoxon non-parametric test. ^&^P < 0.05, ^¥^P < 0.001, P < 0.0001 and ^$^compared delta changes by student t-test. Bold values denotes significant differences at baseline or at pre-post changes at each specific value P < 0.05.*

### Training-Induced Changes in Body Composition Outcomes

After training, in the PreHTN group, there was a significant decrease in body mass (Δ −3.3 kg, *P* < 0.0001), BMI (Δ −1.4 kg/m^2^, *P* < 0.0001), and waist circumference (Δ −2.9 cm, *P* < 0.0001); the NTG also showed similar decreases in body mass (Δ −2.0 kg, *P* < 0.0001), BMI (Δ −0.5 kg/m^2^, *P* < 0.05), and waist circumference (Δ −4.2 cm, *P* < 0.0001). Significant differences in waist circumference (Δ −1.3 cm, *P* = 0.037) were observed between groups (Table 1). Subcutaneous skinfold measurements, including tricipital (Δ −4.6 mm, *P* < 0.05 and Δ −4.8 mm, *P* < 0.05), suprailiac (Δ −7.3 mm, *P* < 0.001 and Δ −5.6 mm, *P* < 0.001), and abdominal (Δ −11.5 mm, *P* < 0.0001 and Δ −7.6 mm, *P* < 0.0001) skinfold measurements, were similarly decreased in the PreHTN group and NTG. Significant differences between groups were detected only in abdominal skinfold thickness (Δ −3.9 mm, *P* < 0.001) (Table 1). The % fat mass was reduced in both the PreHTN group and NTG (Δ −5.8%, *P* < 0.0001; and Δ −3.8%, *P* < 0.001) (Table 1). The % muscle mass was not different between groups (Table 1).

### Training-Induced Changes in Cardiovascular Outcomes

SBP was significantly decreased in the PreHTN group (Δ −8 mmHg, *P* < 0.0001) (Table 1); in contrast, the NTG showed no changes in SBP (Table 1). There was a significant difference between groups in the change in SBP (Δ −8 vs. + 3.0 mmHg, *P* < 0.003) (Table 1). DBP was significantly decreased in the PreHTN group (Δ −5.8 mmHg, *P* < 0.0001) compared to the NTG, and there was a significant difference in the change in DBP (−5.8 vs. −2.0 mmHg, *P* = 0.007) between groups (Table 1). A significant reduction in heart rate at rest was detected in the PreHTN group (Δ −4 beats/min, *P* < 0.0001) but not in the NTG (Table 1).

### Training-Induced Changes in Metabolic Outcomes

Fasting glucose was significantly decreased in both the PreHTN group and NTG (Δ −3.3 mg/dL, *P* < 0.05 and Δ −5 mg/dL, *P* < 0.001), with significant differences between groups (Δ 1.7 mg/dL, *P* < 0.01) (Table 1). TC and LDL-C were significantly decreased in the NTG (Δ −14 mg/dL and Δ −10 mg/dL, *P* < 0.001) (Table 1). There were significant differences in the changes in LDL-C (Δ + 7.4 mg/dL *P* = 0.01) between groups (Table 1). HDL-C was significantly increased (Δ + 5.0 mg/dL, *P* < 0.001) in the PreHTN group, while the NTG showed no significant changes in HDL-C (Table 1). TG levels were significantly reduced in both the PreHTN group and NTG (Δ −13.9 mg/dL, *P* < 0.05 and Δ −22 mg/dL, *P* < 0.0001), with significant differences between groups (Δ 8.1 mg/dL, *P* = 0.045) (Table 1).

### Training-Induced Changes in Performance Outcomes

There were no changes in the 1RM_LE_ strength test in either group (Table 1). However, there were significant improvements in endurance performance, with decreases in the time spent on the 2KMWT in both the PreHTN group and NTG (Δ −3.14 min and Δ −3.34 min, *P* < 0.0001) (Table 1).

### Prevalence of NRs After HIIT Exercise Training

There were significant differences in the prevalence of NRs in the PreHTN group vs. the NTG based on the following variables: BMI (13.6 vs. 40.0%, *P* = 0.006), abdominal skinfold (6.8 vs. 15.0%, *P* < 0.0001), LDL-C (72.7 vs. 50.0%, *P* = 0.032), HDL-C (56.8 vs. 77.5%, *P* = 0.045), and TG (70.5 vs. 35.0%, *P* < 0.001) (Table 2). The risk (based on OR: 95% CI) of no response was high (≥2-fold) in the PreHTN group for LDL-C (OR 2.6: 1.0 to 6.6) and TG (OR 4.4: 1.7 to 11) (Table 2).

**Table 2:** Prevalence of non-responders by health status (i.e., prehypertensive and normotensive subjects) after intervention.

	Response	PreHTN	NTG	OR (95% IC)	PreHTN vs. NTG *X*^2^
**Anthropometry**					
	Rs	88.6 (39)	77.5 (31)		
Body mass, %/(*n*=)	NRs	11.4 (5)	22.5 (9)	0.4 (0.1 to 1.4)	*P* = 0.171
Body mass index, %/(*n*=)	NRs	13.6 (6)	40.0 (16)	0.2 (0.08 to 0.6)	***P* = 0.006**
	Rs	86.4 (38)	60.0 (24)		
Waist circumference, %/(*n*=)	NRs	4.5 (2)	15.0 (6)	0.2 (0.05 to 1.4).	*P* = 0.103
	Rs	95.5 (42)	85.0 (34)		
Tricipital skinfold, %/(*n*=)	NRs	9.0 (4)	0 (0)	0.8 (0.2 to 3.2)	*P* = 0.255
	Rs	90.9 (40)	100 (40)		
Supra-iliac skinfold, %/(*n*=)	NRs	11.3 (5)	12.5 (5)	0.5 (0.2 to 1.8)	*P* = 0.331
	Rs	88.6 (39)	87.5 (35)		
Abdominal skinfold, %/(*n*=)	NRs	6.8 (3)	15.0 (6)	1.5 (0.9 to 3.2)	***P* < 0.0001**
	Rs	93.1 (41)	85.0 (34)		
Fat mass, %/(*n*=)	NRs	20.4 (9)	20.0 (8)	0.6 (0.1 to 1.1)	*P* = 0.651
	Rs	79.5 (35)	80.0 (32)		
Muscle mass, %/(*n*=)	NRs	97.7 (43)	100 (40)	0.1 (0.1 to 0.9)	*P* = 0.288
	Rs	2.2 (1)	0 (0)		
**Cardiovascular**					
Heart rate rest, %/(*n*=)	NRs	29.5 (13)	37.5 (15)	0.7 (0.2, 1.7)	*P* = 0.322
	Rs	70.4 (31)	62.5 (25)		
**Metabolic**					
Fasting glucose, %/(*n*=)	NRs	65.9 (29)	70.0 (28)	0.8 (0.3 to 2.0)	*P* = 0.668
	Rs	34.1 (15)	30.0 (12)		
Total cholesterol, %/(*n*=)	NRs	61.4 (27)	52.5 (21)	1.4 (0.6 to 3.4)	*P* = 0.412
	Rs	38.6 (17)	47.5 (19)		
Low-density lipids, %/(*n*=)	NRs	72.7 (32)	50.0 (20)	2.6 (1.0 to 6.6)^#^	***P* = 0.032**
	Rs	27.3 (12)	50.0 (20)		
High-density lipids, %/(*n*=)	NRs	56.8 (25)	77.5 (31)	0.3 (0.1 to 0.9)	***P* = 0.045**
	Rs	43.2 (19)	22.5 (9)		
Triglycerides, %/(*n*=)	NRs	70.5 (31)	35.0 (14)	4.4 (1.7 to 11.0)^#^	***P* = 0.001**
	Rs	29.5 (13)	65.0 (26)		
**Strength performance**					
1RM_LE_, %/(*n*=)	NRs	54.5 (24)	67.5 (27)	0.3 (0.1 to 1.8)	*P* = 0.466
	Rs	45.4 (20)	32.5 (13)		
**Strength performance**					
2KMWT, %/(*n*=)	NRs	15.9 (7)	25.0 (10)	0.9 (0.5, 2.5)	*P* = 0.121
	Rs	84.0 (37)	75.0 (30)		

*PreHTN, pre hypertensive group; NTG, healthy group; 1RM_LE_, one maximum repetition strength test; 2KMWT, 2 kilometers walking test; OR, odds ratios (95% IC). ^#^High risk (≥2 fold) to suffer a non-response. Bold values denote significant difference in the NRs prevalence between groups at level P < 0.05.*

**FIGURE 3:**
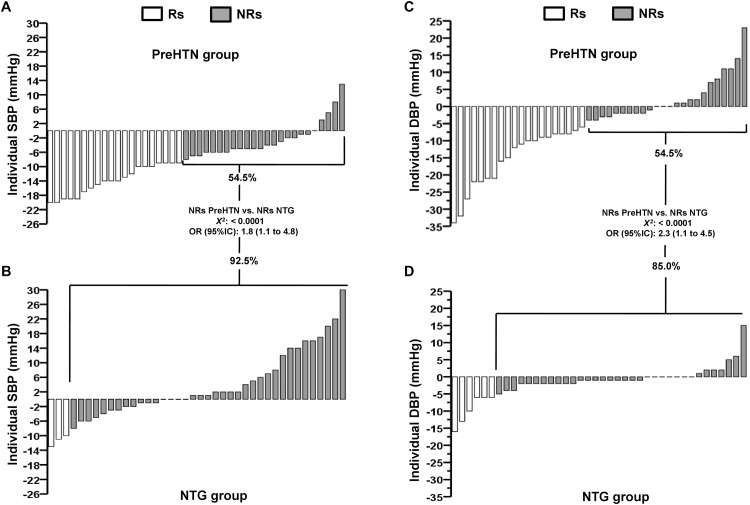
Individual changes for systolic blood pressure in **(A)** prehypertensive and **(B)** normotensive, and individual changes for diastolic blood pressure in **(C)** prehypertensive and **(D)** normotensive subjects after 16-weeks of HIIT. PreHTN, prehypertensive group; NTG, normotensive subjects; SBP, systolic blood pressure; DBP, diastolic blood pressure; Rs, responders; NRs, non-responders; OR, odds ratios.

Figure 3A shows the delta values for individual changes in SBP (ΔSBP in mmHg) in the PreHTN group, in which the prevalence of NRs was 54.5% (24 patients). Figure 3B shows the delta values for individual changes (ΔSBP in mmHg) in the NTG, in which the prevalence of NRs was 92.5% (37 patients). There was a significant difference in the prevalence of NRs based on SBP between the PreHTN group and NTG (54.5 vs. 92.5%, *P* < 0.0001) (Figures 3A,B).

Figure 3C shows the delta of individual changes in DBP (ΔDBP in mmHg) in the PreHTN group, in which the prevalence of NRs was 54.5% (24 patients). Figure 3D shows the delta of individual changes in DBP (ΔDBP in mmHg) in the NTG, in which the prevalence of NRs was 85.0% (34 patients). There was a significant difference in the prevalence of NRs based on DBP between the PreHTN group and NTG (54.5 vs. 85.0%, *P* < 0.0001) (Figures 3A,D).

Table 3 shows the five models used to predict a response in SBP, where model 3 (based on baseline SBP + body composition and cardiovascular measurements), model 2 (based on baseline SBP + body composition measurements), and model 1 (based on only baseline SBP), which we named previously as ‘health status,’ could explain, respectively, 53.8, 52.3, and 51.2% of the total variance in SBP changes after training. Model 5, in which performance variables were added, explained a significant 25.5% of the total variance in SBP changes (Table 3).

**Table 3:** Percentage of total variance explained by 5 models of factors predictive SBP decreases.

	Variables included in the model	R	Total model R^2^	% Variance explained by the model	*P*-value
Model 1	Baseline SBP (mmHg)	0.715	0.512	51.2%¶	***P* < 0.0001**
Model 2	Baseline SBP (mmHg)	0.723	0.523	52.3%¶	***P* < 0.0001**
	+*Body composition*; Δbody mass (kg), Δwaist circumference (cm), Δtricipital skinfold (mm), Δsupra-iliac skinfold (mm), ΔAbdominal skinfold (mm), Δ % fat mass (%), and Δ % muscle mass (%)				
Model 3	Baseline SBP (mmHg)	0.734	0.538	53.8%¶	***P* < 0.0001**
	+*Body composition*; Δbody mass (kg), Δwaist circumference (cm), Δtricipital skinfold (mm), Δsupra-iliac skinfold (mm), ΔAbdominal skinfold (mm), Δ % fat mass (%), Δ % muscle mass (%), +*Cardiovascular;*Δ heart rate at rest (beats/min)				
Model 4	Baseline SBP (mmHg)	0.435	0.189	18.9%	*P* = 0.072
	+ *Body composition*; Δbody mass (kg), Δwaist circumference (cm), Δtricipital skinfold (mm), Δsupra-iliac skinfold (mm), ΔAbdominal skinfold (mm), Δ % fat mass (%), Δ % muscle mass (%), + *Cardiovascular;*Δ heart rate at rest (beats/min). + *metabolic;*Δ TC, Δ LDL-C, ΔHDL-C, and ΔTG.				
Model 5	Baseline SBP (mmHg)	0.523	0.255	25.5%	***P* < 0.05**
	+ *Body composition*; Δbody mass (kg), Δwaist circumference (cm), Δtricipital skinfold (mm), Δsupra-iliac skinfold (mm), ΔAbdominal skinfold (mm), Δ % fat mass (%), Δ % muscle mass (%), + *Cardiovascular;*Δ heart rate at rest (beats/min) + *metabolic;*Δ TC, Δ LDL-C, ΔHDL-C, ΔTG + *strength and endurance performance;*Δ1RM_LE_ (kg), Δ2KMWT (min.s)				

*SBP, systolic blood pressure; Δ, delta changes to each body composition, cardiovascular, metabolic outcome; TC, total cholesterol, LDL-C, low-density lipids, HDL-C, high-density lipids, TG, triglycerides. Bold values denote significant correlation. ^¶^ denotes the most strongest (>50% variance explained) models predictors of SBP changes after 16-weeks of HIIT.*

## Discussion

This study has two main findings: (*i*) the prevalence of NRs based on SBP, DBP, and other comorbidities is different between groups; and (*ii*) between the 5 tested models (from the most basic to the most complex) for BP reduction, baseline SBP alone (model 1), referred to as ‘BP-health status’ by us, is the simplest model with similar (e.g., model 2; 52.3%, model 3; 53.8%, both *P <* 0.0001) efficacy for predicting Rs with a predictive capacity in SBP (51.2%) after 16 weeks of HIIT, when compared with other more complex models that included added outcomes. Decreases in SBP were accompanied by other known effects of exercise, but not in both cohorts. Accordingly, HIIT decreased both SBP (Δ −8 mmHg) and DBP (Δ −5.8 mmHg) only in prehypertensive subjects, whereas improvements in anthropometric/body composition, metabolic and endurance performance were observed in both groups.

Contrary to our hypothesis, the prevalence of NRs between prehypertensive and normotensive subjects after HIIT was different, whereas changes in anthropometric, metabolic, and endurance performance were almost similar. However, there is little evidence at present regarding the prevalence of NRs in terms of changes in SBP/DBP after HIIT (Higgins et al., 2015; Alvarez et al., 2017). Including other training methods, such as endurance or resistance training (Bouchard et al., 2012; Moker et al., 2014), would help confirm which training modality is more appropriate for decreasing BP with fewer NRs. For example, in ∼1600 subjects after 20 weeks of endurance training (30–50 min/session, 3 days/week, 55–75% of the maximum oxygen uptake), 12.2% of subjects were considered NRs based on decreased SBP (Bouchard et al., 2012). After 6 months of endurance (65–80% peak of oxygen uptake, walking/jogging), resistance (8–12 repetitions, 8 exercises, 70–85% of one-maximum repetition, 3 days/week) or combined training, ∼60.9% of subjects were NRs based on decreased SBP and ∼59.1% of subjects were NRs based on decreased DBP (Moker et al., 2014). Furthermore, 6 weeks of HIIT in adults (3 × 1 min maximum intensity with 2 min recovery, 3 days/week) resulted in ∼61.5% NRs in terms of decreased DBP (Higgins et al., 2015). We found that among prehypertensive subjects, 11.4% were NRs for decreased SBP and 31.8% were NRs for decreased DBP. By contrast, the prevalence of NRs based on decreased SBP and DBP in the NT group was greater at 68.8 and 35.0%, respectively. Additionally, among 23 healthy adults who underwent 6 weeks of HIIT (Higgins et al., 2015) a wide interindividual variability was reported, in which Rs showed a decrease in SBP of ∼10 mmHg, and NRs showed an increase in SBP of 10 mmHg.

Interestingly, in terms of BP effects, 30 min of moderate-intensity exercise/day in combination with resistance training for a cumulative 150 min/week decreased BP by 5–7 mmHg in individuals with HTN (Pescatello et al., 2015). Here we report that 16 weeks of HIIT at a low volume of exercise/week (∼21–30 min/week) is able to decrease BP (SBP −8 mmHg and DBP −5.8 mmHg) in a prehypertensive cohort to similar values as for endurance or resistance training, but with less time investment. The evidence supporting endurance exercise for normalizing BP is not novel (Cade et al., 1984); however, to the best of our knowledge, the evidence for HIIT in decreasing BP has been little explored at all in prehypertensive or hypertensive populations. After 12 weeks of HIIT and endurance training, HIIT decreased the average 24-h ambulatory SBP of hypertensive subjects by 12 mmHg, whereas endurance training decreased it by only 4.5 mmHg (Molmen-Hansen et al., 2012). Similarly, the ambulatory 24-h DBP was decreased by 8 mmHg after HIIT and by 3.5 mmHg after endurance training, leading the authors to conclude that the decrease in BP among patients with HTN is intensity-dependent. In another study, after 16 weeks of HIIT, SBP and DBP were decreased in hypertensive patients by 6 and 4 mmHg, respectively, but these measures were not markedly changed in normotensive patients, with decreases of ∼1 mmHg for both variables (Guimaraes et al., 2010), which is similar to our findings. Moreover, after 12 weeks of HIIT in healthy men, SBP decreased by 18 mmHg (Nybo et al., 2010). Thus, it is not surprising that short HIIT programs (Whyte et al., 2010) lasting only 2 weeks showed decreases in SBP of 6 mmHg and in DBP of 9 mmHg among prehypertensive individuals. The mechanisms by which HIIT leads to decreases in BP have not been fully elucidated, but it has been reported that a combination of factors could be involved, including increasing shear stress, decreasing sympathetic nervous activity, reducing vascular peripheral resistance, and increasing nitric oxide-mediated vasodilatation (Halliwill, 2001). In our study, although the BP increases at the individual level were high in both groups, they were higher in the NT group than in the PreHTN group (Figure 2). A report from an epidemiological study has shown increases in SBP of ∼10 mmHg as an adverse response to exercise (Bouchard et al., 2012). Here, we report, using an experimental approach, increases in SBP by ∼30 to 15 mmHg in the normotensive group and by 14 to 20 mmHg in the HTN group, indicating the relevance of reporting data not only as the ‘mean,’ but also at the interindividual level using a typical sample size (∼20–40 subjects) for experimental studies.

In our study, other anthropometric/body composition effects from HIIT included body mass decreases (−3.3 and −2.0 kg), BMI (−1.4 and −0.5% kg/m^2^), tricipital (−4.6 and −4.8 mm), and suprailiac (−7.3 and −5.6 mm) skinfold thickness, and fat mass (−5.8 and −3.8%), which were similar in both the prehypertensive and hypertensive groups. Other authors have reported similar results after 2 weeks of HIIT (Whyte et al., 2010; Boutcher, 2011), and these findings were corroborated with molecular changes after HIIT (Little et al., 2011). For example, in the study of Whyte et al. (2010), 6 sessions of 30 s of ‘all out’ exercise were shown to decrease body mass by 1 kg and waist circumference by 2.4 cm. In our previous study using 16 weeks of HIIT, we found a 1.6 kg decrease in body mass, 4.1 cm decrease in waist circumference, and ∼20% decrease in subcutaneous fat in patients with type 2 diabetes (T2DM) (Alvarez et al., 2016). Other authors, however, have not observed decreases in body mass after 12 months of HIIT in adolescents (Tjønna et al., 2009). Additionally, in the present study, for some variables we found significant differences between groups in the magnitude of change, including waist circumference (∼1 cm, *P* = 0.037) and abdominal skinfolds (∼4 mm, *P* < 0.0001). We presume that these effects would be in addition to a similar general effect of HIIT. Fat mass, for example, was decreased similarly between groups; thus, we speculate that the same specific (not measured) molecular mechanisms accrued as a result of the HIIT protocol, and that any small differences in the magnitude of changes between groups may have been influenced by the anthropometric and/or the BP differences between groups at baseline (pre-HIIT). The weight loss, and other fat markers such as waist circumference and subcutaneous skinfold thickness, as well as postexercise adrenergic mechanisms, are relatively well known and described (Boutcher, 2011).

Fasting glucose was decreased after HIIT in both groups, with a more pronounced decrease in the NT group (∼5 mg/dL) over the PreHTN group (∼3.3 mg/dL). Our previous study reported that 16 weeks of HIIT resulted in a decrease in fasting glucose of ∼15% in T2DM patients (Alvarez et al., 2016), showing more pronounced benefits in the same time period than in our present non-diabetic sample. However, we also reported in this study that there were decreases in TG in both groups, as well as TC and LDL-C in the NT group, and increases in HDL-C in the prehypertensive cohort. Interestingly, TC, LDL-C, and TG were decreased by a greater magnitude in the NT group than in the PreHTN group. The additional metabolic benefits of HIIT, including improving dyslipidemia, alongside the benefits on BP are considered relevant for decreasing/preventing comorbidities in prehypertensive populations. Reduced risk of HTN in populations with dyslipidemia is related to higher physical activity levels than the minimal recommended activity level in current guidelines (Williams and Franklin, 2015). In the present study, the HIIT program had a weekly time commitment of ∼60 to ∼90 min divided into three exercise sessions (∼21–30 min/session), which was lower than the minimum 150 min/week of activity recommended in current guidelines (O’Donovan et al., 2010). For example, in T2DM patients, decreases of 2.1/0.9 mmHg in BP reduced the risk of major cardiovascular events by 10% (Turnbull et al., 2005), whereas the risk of developing coronary artery disease was reduced by 2–3% for each 1 mg/dL increase in HDL-C (Maron, 2000). Thus, the 8 mmHg reduction in systolic BP and the ∼5 mg/dL increase in HDL-C observed in prehypertensive women in the present study may have clinical implications. The mechanisms by which HIIT decreases plasmatic lipoprotein levels are unclear, but we presume that a decrease in the intramyocellular fat in the liver could play a role (Heijden et al., 2010).

A similar 16-week HIIT program was shown to improve endurance performance, similarly decreasing the time needed to complete the 2KMWT in T2DM patients by 2 min (Alvarez et al., 2016). There is strong evidence that HIIT increases endurance performance (Gibala et al., 2012), which is corroborated by the findings in our prehypertensive cohort. Twelve weeks of HIIT have been reported to increase the maximum oxygen uptake ∼13%, similar/or more than traditional endurance training of ∼7%, with this outcome considered as a performance marker frequently related with health and disease (Nybo et al., 2010).

Multiple regressions analyses of baseline SBP (model 1) and baseline SBP associated with one (anthropometric, model 2), two (cardiovascular, model 3), three (metabolic, model 4), and four (performance, model 5) additional parameters showed that baseline SBP (model 1) explained a similar percentage of variance (51.2%) to that of the more complex models tested (model 2: 52.3%; model 3: 53.8%; and model 5: 25.5%). Thus, our findings confirm that baseline measurements can be useful for predicting responses to HIIT. More recently, the ‘magnitude’ of the hypotensive effect has been reported as a predictive factor for decreasing BP after chronic exercise (Hecksteden et al., 2013b). Unfortunately, the authors referred to 4 weeks of training as ‘chronic’ exercise, and these results are limited to the specific endurance protocol used, showing the importance of effects, the prevalence of NRs, and predictive factors of a response to long-term HIIT.

The strengths of the present study include our data on the effects, prevalence of NRs and predictive factors for decreasing BP in prehypertensive subjects. One limitation was the lack of a true no-exercise control group. Another limitation was that we used BIA to assess body composition variables; however, BIA is not considered the ‘gold standard’ method. We also did not implement dietary control during the intervention. Nonetheless, we continually reminded subjects to maintain their baseline dietary habits. Finally, as heart rate at rest, fasting glucose, HDL-C, and 1RM_LE_ were almost significantly different at baseline, the significant differences in each group pre- and post-intervention (Table 1) must be interpreted with caution.

## Conclusion

In conclusion, the prevalence of NRs based on SBP and DBP was different between prehypertensive and normotensive subjects after 16 weeks of HIIT. Other comorbidities such as body composition and metabolic outcomes showed almost similar modifications between prehypertensive and normotensive subjects, being the most basic predictive factor for BP reduction baseline SBP, which we refer to as ‘BP health status’ (51.2%). This improvement in BP was accompanied by other known improvements of HIIT on body composition, metabolic, and endurance performance in both study cohorts.

## Novelty and Significance

### What Is New?

Although both prehypertensive and normotensive groups showed improvements in fat markers, metabolic risk factors (fasting glucose, lipid profile), and endurance performance, and thus a decrease in comorbidities, there was a different prevalence of non-responders based on decreased systolic and diastolic BP among prehypertensive individuals compared to normotensive individuals.

### What Is Relevant?

To normalize high blood pressure and improve lipid profiles, an appropriate type of exercise training must be chosen. In addition, other non-pharmacological strategies are required to prevent hypertension.

### Summary

Among participants in a prehypertensive state, altered blood pressure alone (health status) is a powerful predictive factor for the normalization of blood pressure, an increase in endurance performance and improvements in other metabolic risk factors after exercise.

## Availability of Data and Material

The datasets during and/or analyzed during the current study available from the corresponding author on reasonable request.

## Ethics Statement

Prior to providing written informed consent, the clinical records of all participants were reviewed to ensure that they met all of the inclusion criteria. Additionally, all subjects were personally informed about the study procedures that would occur before, during and after the intervention. All procedures were approved by the local Ethics Committee of the Family Healthcare Center Tomás Rojas in the city of Los Lagos, Chile.

## Author Contributions

CÁ and RR-V conceived and designed the project. CÁ and RR-C reviewed the literature studies and conducted the data extraction. CÁ conducted the data analyses. CÁ, RR-C, and MI were responsible for the data interpretation. CÁ, RRC, and CC-M drafted the manuscript. RR-V and MI revised it critically for the intellectual contributions. CÁ and RR-C coordinated the study development. All authors reviewed, edited, read, and approved the final manuscript.

## Conflict of Interest Statement

The authors declare that the research was conducted in the absence of any commercial or financial relationships that could be construed as a potential conflict of interest.

## Abbreviations

1RM_LE_: one-maximum repetition test of leg extension
2KMWT: 2 kilometer walking test
BMI: body mass index
DBP: diastolic blood pressure
HDL-C: high-density lipoprotein
HIIT: high-intensity interval training
LDL-C: low-density lipoproteins
NRs: non-responders
NTG: normotensive group
OR: odds ratios
PreHTN: prehypertensive group
Rs: responders
SBP: systolic blood pressure
TC: total cholesterol
TG: triglycerides.
